# Phase I dose-escalation study to determine the safety, pharmacokinetics and pharmacodynamics of brivanib alaninate in combination with full-dose cetuximab in patients with advanced gastrointestinal malignancies who have failed prior therapy

**DOI:** 10.1038/bjc.2011.182

**Published:** 2011-05-31

**Authors:** C R Garrett, L L Siu, A El-Khoueiry, J Buter, C M Rocha-Lima, J Marshall, P LoRusso, P Major, J Chemidlin, O Mokliatchouk, L Velasquez, W Hayes, D Feltquate, S Syed, S Ford, G Kollia, S Galbraith, D S A Nuyten

**Affiliations:** 1 Department of Gastrointestinal Oncology, Unit 426, The University of Texas MD Anderson Cancer Center, 1515 Holcombe Boulevard, Houston, TX 77030-4009, USA; 2 Division of Medical Oncology and Hematology, Princess Margaret Hospital, Toronto, Ontario, Canada; 3 Division of Medical Oncology, USC Norris Cancer Center, Los Angeles, CA, USA; 4 Department of Medical Oncology, VU University Medical Center, Amsterdam, The Netherlands; 5 Department of Hematology/Oncology, Sylvester Comprehensive Cancer Center, University of Miami School of Medicine, Miami, FL, USA; 6 Division of Hematology/Oncology, Lombardi Comprehensive Cancer Center, Georgetown University Hospital, Washington, DC, USA; 7 Phase I Clinical Trials Program, Karmanos Cancer Institute, Wayne State University, Detroit, MI, USA; 8 Division of Medical Oncology, The Margaret and Charles Juravinski Cancer Centre, Hamilton, Ontario, Canada; 9 Bristol-Myers Squibb, Princeton, NJ, USA

**Keywords:** antiangiogenesis, brivanib, cetuximab, gastrointestinal tumours

## Abstract

**Background:**

The objectives of this phase I study were to determine the safety, pharmacokinetics (PK), pharmacodynamics and efficacy of brivanib combined with full-dose cetuximab in patients with advanced gastrointestinal malignancies.

**Methods:**

Patients with advanced gastrointestinal malignancies who had failed prior therapies received brivanib (320, 600 or 800 mg daily) plus cetuximab (400 mg m^–2^ loading dose then 250 mg m^–2^ weekly). Assessments included adverse events, PK, tumour response, 2[18F]fluoro-2-deoxyglucose positron-emitting tomography and *K-Ras* mutation analyses.

**Results:**

Toxicities observed were manageable; the most common treatment-related toxicities (>10% of patients) were fatigue, diarrhoea, anorexia, increase in aspartate aminotransferase and alanine aminotransferase, acneiform dermatitis, headache, mucosal inflammation, nausea, dry skin, vomiting, hypertension, pruritus, proteinuria and weight loss. Of 62 patients, 6 (9.7%) had objective radiographic partial responses, with an overall response rate of 10%. Median duration of response was 9.2 months; median progression-free survival was 3.9 months.

**Conclusions:**

The acceptable toxicity profile and efficacy of brivanib observed in this study were promising. These findings are being further evaluated in a phase III study of brivanib plus cetuximab *vs* cetuximab alone in patients previously treated with combination chemotherapy for *K-Ras* wild-type advanced metastatic colorectal cancer.

Angiogenesis is critical for growth, metastasis and invasion of tumours (Kerbel, 2008). Vascular endothelial growth factor (VEGF) is the major mediator of angiogenesis, although multiple other angiogenic factors such as fibroblast growth factor-1 (FGF-1, aFGF) and -2 (FGF-2, bFGF) and platelet-derived growth factor are involved in the initiation and maintenance of angiogenesis and tumourigenesis (Kerbel, 2008). Bevacizumab, which inhibits VEGF receptor-2 (VEGFR-2) signalling through inhibition of VEGF-A ligand binding, prolongs survival in patients with metastatic colorectal cancer in both the first- and second-line settings when used in combination with cytotoxic chemotherapy; however, observed responses are transient, with a median overall survival of 12.9 months for patients receiving second-line treatment and 23 months for those receiving first-line treatment (Kabbinavar *et al*, 2003; Hurwitz *et al*, 2004; Giantonio *et al*, 2007).

Cetuximab, a chimeric monoclonal antibody targeting the epidermal growth factor receptor (EGFR), has demonstrated efficacy and overall survival benefits in patients with *K-Ras* wild-type metastatic colorectal cancer (Cunningham *et al*, 2004; Sobrero *et al*, 2008). Preclinical studies suggest that VEGF overexpression has a role in acquired resistance to anti-EGFR therapy (Bianco *et al*, 2008) and that dual inhibition of VEGF and EGF signalling may overcome such resistance (Naumov *et al*, 2009). It is unclear whether these observations translate into prolonged clinical benefit.

Upregulation of alternate proangiogenic signals, such as the FGF signalling pathway, is one of the proposed mechanisms in the development of resistance to VEGF-directed antiangiogenic therapy (Bergers and Hanahan, 2008). Serum bFGF levels have been shown to be increased following first-line treatment of patients with metastatic colorectal cancer treated with combination chemotherapy and monoclonal anti-VEGF antibody therapy (Kopetz *et al*, 2009). Targeting FGF, VEGF and EGF signalling pathways simultaneously may provide additional clinical benefit to patients with advanced gastrointestinal malignancies. Brivanib is the first orally bioavailable selective dual inhibitor of FGF and VEGF signalling and is administered as brivanib alaninate, the L-alanine ester prodrug of the active moiety brivanib. In preclinical studies, brivanib has shown strong antiangiogenic and antitumour effects on tumour cells across a range of tumour types, including colon, breast, liver and lung (Bhide *et al*, 2006, 2010; Huynh *et al*, 2008), and no evidence of evasive resistance when administered to RIP-Tag2/Bl6 mice (Allen *et al*, 2009). In a recent phase II study in advanced/metastatic hepatocellular carcinoma, brivanib demonstrated encouraging efficacy, with an overall survival of 10 months, and was generally well tolerated (Park *et al*, 2011). Cetuximab has been combined with anti-VEGF monoclonal antibody therapy (bevacizumab) safely with preliminary suggestion of improved efficacy (Saltz *et al*, 2007); however, large, randomised phase III studies failed to show a survival benefit associated with cetuximab or panitumumab when added to bevacizumab plus cytotoxic chemotherapy (Hecht *et al*, 2009; Tol *et al*, 2009). The FGF pathway is a known alternate angiogenesis pathway to the VEGF pathway and may account for the resistance to anti-VEGF therapy (Kopetz *et al*, 2009); thus a combined VEGF and FGF receptor inhibitor holds the promise of superior efficacy when used in combination with an EGFR pathway inhibitor.

The primary goals of the current study were to (a) define a dose for combination treatment with brivanib alaninate and cetuximab for further evaluation in phase II/III studies, (b) develop knowledge about dose-limiting toxicity (DLT) and (c) assess the preliminary efficacy and safety of this combination therapy in patients with advanced gastrointestinal malignancies.

## Materials and methods

### Patients

Patients were required to have the following characteristics: age ⩾18 years; Eastern Cooperative Oncology Group performance status score 0 or 1; histologic or cytologic diagnosis of gastrointestinal malignancy (if the patient entered during the dose-expansion phase, they had to have tissue-verified colorectal cancer); tumour biopsy tissue available for correlative biomarker analysis (*K-Ras* testing on tissue was performed *post hoc* after trial completion); radiographic or tissue confirmation that the disease was locally advanced/metastatic; measurable disease; adequate bone marrow, hepatic and renal function; toxicity related to prior therapy had to be resolved to baseline or deemed irreversible; at least 4 weeks had to pass since last chemotherapy, immunotherapy, radiotherapy, anticancer hormonal therapy or targeted therapy, and at least 6 weeks since last therapy with bevacizumab, nitrosoureas, mitomycin C and/or liposomal doxorubicin; and women of child-bearing age had to have a negative pregnancy test. Prior anti-EGFR therapy and anti-VEGF monoclonal antibody therapy were allowed. Patients who had prior treatment with VEGFR-tyrosine kinase inhibitors were ineligible. A DLT was defined, for the purposes of this study, as any of the following events occurring in the first 4 weeks of study treatment: grade 4 neutropenia (i.e. absolute neutrophil count (ANC) <500 cells mm^–3^ for 5 or more consecutive days) or febrile neutropenia (i.e. fever >38°C with an ANC <500 cells mm^–3^ requiring hospitalisation); grade 4 thrombocytopenia or bleeding episode requiring platelet transfusion; grade 3 nausea and/or emesis despite the use of maximal medical intervention; grade 2 or greater cardiovascular toxic effect; any grade 3 or greater nonhaematologic toxic effect; or delayed recovery (2 weeks or more) after scheduled re-treatment from a delayed toxic effect related to treatment with cetuximab and brivanib.

### Study design

This was an open-label, phase I study of brivanib alaninate administered orally in combination with intravenous cetuximab to patients with advanced gastrointestinal malignancy. This study was conducted in accordance with good clinical practice, as defined by the International Conference on Harmonization and in accordance with the ethical principles underlying European Union Directive 2001/20/EC and the United States Code of Federal Regulations, Title 21, Part 50 (21CFR50). The protocol, amendments and patient-informed consent received appropriate approval by the respective Institutional Review Board/Independent Ethics Committees prior to study initiation. Informed consent was obtained from each patient prior to study participation.

The primary objective was to assess the DLT of brivanib alaninate in combination with cetuximab and to define the maximum tolerated dose (MTD) in patients with advanced gastrointestinal malignancy who had failed prior therapy.

Secondary objectives included assessment of radiographic evidence of antitumour activity, evaluation of changes by 2[18F]fluoro-2-deoxyglucose positron-emitting tomography (FDG-PET) scan and/or radiologic response as defined by the modified World Health Organisation (WHO) criteria, duration of response, duration of disease control and time to progression at doses other than the MTD. Additional FDG-PET-specific objectives were to assess the tumour metabolic response and the association of tumour metabolic changes with clinical outcome (progression-free survival; PFS) in this study population and to assess the reproducibility of FDG-PET measurements of standardised uptake value (SUV) parameters in this multicentre trial. Additional secondary objectives were to determine the disease control rates, duration of response, duration of disease control and PFS based on the modified WHO criteria in response-evaluable patients at the MTD, and to assess the pharmacokinetics (PK) of brivanib alaninate when administered in combination with cetuximab.

A *post hoc* additional exploratory biomarker analysis to evaluate the relationships between *K-Ras* mutation status and efficacy end points in patients with colorectal cancer was performed.

### Treatment

On cycle 1, day 1 of a 28-day treatment cycle, a single dose of brivanib alaninate was administered, followed by a 6-day washout period. On cycle 1, day 8, continuous daily oral dosing of brivanib alaninate was started together with a single loading dose of intravenous cetuximab 400 mg m^–2^ infused over 120 min. Beginning on cycle 1, day 15, cetuximab was administered weekly at 250 mg m^–2^, infused over 60 min. For the remainder of the study, patients received oral brivanib alaninate on a daily continuous schedule and intravenous cetuximab on a weekly basis. Dose escalation of brivanib alaninate starting at 320 mg with two additional escalations of 600 and 800 mg was explored (see Appendix A for further treatment details).

### Assessments

#### Safety

Adverse events (AEs) were evaluated according to the National Cancer Institute Common Terminology Criteria for Adverse Events (v 3.0) on a continuous basis, while the patient was on study and until ⩽30 days after the last dose of study drug or until all treatment-related AEs had recovered to baseline or were deemed irreversible. Once a subject had been off treatment due to toxicity, assessments were to be made every 28 days until all study-related toxicities resolved to baseline, stabilised or were deemed irreversible.

#### Pharmacokinetics

Brivanib alaninate is rapidly converted to brivanib (the active moiety) *in vivo*. Hence, only the active moiety was measured in plasma. Plasma samples for determination of brivanib concentrations were drawn pre-dose and serially (15, 30 and 45 min, and 1, 2, 4, 6, 8 and 24 h after dosing) after dosing on cycle 1, day 1 and cycle 1, day 8. Serum samples for determination of cetuximab concentrations were drawn pre-dose and serially (2, 4, 6 and 24 h after dosing) after dosing on cycle 1, day 8. Serum samples for both brivanib and cetuximab were also drawn pre-dose on cycle 1, day 15 and cycle 2, day 1. For analyses of brivanib and cetuximab concentrations in serum and plasma samples, see Appendix B.

#### Efficacy

Tumour response was determined using modified WHO criteria (see Appendix C).

#### FDG-PET and K-Ras mutations analyses

See Appendix D.

### Statistical methods

Cohorts of 3–10 patients were to be treated at each dose level until the MTD was defined based on observance of DLTs; however, the MTD was not reached due to lack of DLT. Thus, at the maximum dose level (MDL) of 800 mg, a total of 40 response-evaluable patients were accrued in two stages, with the number of patients at each stage based on a modified Gehan two-stage approach (see Appendix E for further details on statistical analyses).

## Results

### Patient disposition and demographics

Eighty-five patients were enrolled on this study, 62 of whom were treated with brivanib alaninate (Table 1) and 61 of whom received brivanib alaninate plus cetuximab (24 of the 85 patients failed screening). The majority of patients were males (61% *vs* 39%); median age was 60 years. Fifty-nine patients had colorectal cancer, two had oesophageal cancer and one had fibrolamellar hepatocellular carcinoma. The most common metastatic sites were liver (53 patients), lung (51 patients) and lymph node (19 patients).

### Safety

The MTD was not formally reached and the expansion cohort was opened at the predefined MDL based on the MTD (800 mg) reached in the phase I brivanib monotherapy study (CA182002) (Jonker *et al*, 2010). One patient had a DLT (bilateral pulmonary embolism) considered possibly related to treatment with brivanib alaninate at a dose of 320 mg combined with cetuximab. The event did not lead to any further complications, but the patient died 3 weeks later due to disease progression. No further DLTs occurred at any dose.

Overall, brivanib treatment-related AEs were acceptable when administered daily at doses of 320, 600 and 800 mg in combination with cetuximab (Table 2). Six patients (9.7%), all of whom were in the 800-mg cohort, had grade 1/2 palmar–plantar dysesthesias.

The majority of treatment-related AEs were grade 1/2 in severity. The most frequently reported treatment-related grade 3/4 AEs (>5% of patients) were fatigue and increase in hepatic transaminases. The incidence of grade 3/4 palmar–plantar dysesthesias was infrequent, with only one reported case in the 800-mg cohort (1.6% of patients). In total, 14 patients (22.6%) reported serious (grade 3/4) treatment-related AEs during the study, including vomiting, dehydration and pyrexia. Seven patients discontinued due to study drug-unrelated AEs, including small intestinal obstruction, vomiting, pneumonia, left ventricular dysfunction, hyperbilirubinaemia, drug hypersensitivity to cetuximab, disease progression, angio-oedema and hypertension in one patient each. In addition, pneumonia and malignant neoplasm progression occurred in the same patient. Four patients discontinued due to drug-related toxicities, which included sepsis, aspartate aminotransferase elevation, dehydration and angio-oedema. All AEs leading to study discontinuation occurred in the 800-mg cohort.

Twenty patients had at least one dose reduction, including two in the 600-mg cohort and 18 in the 800-mg cohort. No patient in the 320-mg cohort had a dose reduction. Dose reductions due to cetuximab-related AEs occurred across all cohorts in 16 patients (26%), two in the 600-mg cohort and 14 in the 800-mg cohort. Reasons for dose reduction were delayed nonhaematologic recovery (five patients, 8%), skin toxicity (four patients, 6%) and delayed haematologic recovery (one patient, 2%).

In all, 15 patients died during the study, 14 due to disease progression and 1 due to study drug toxicity. One patient, who was treated with brivanib alaninate 800 mg in combination with cetuximab, died on day 47 due to sepsis related to rectal perforation. Both events were considered possibly related to treatment.

### Pharmacokinetics

At the MDL, cetuximab had no effect on brivanib maximum plasma concentration (*C*_max_) or area under the plasma concentration–time curve extrapolated to infinity (AUC(INF)): 90% CIs for the *C*_max_ and AUC(INF) geometric mean ratios with and without cetuximab were within the 80–125% interval (Table 3).

### Efficacy

Six patients (9.7%) achieved a partial response, all of whom were in the brivanib alaninate 800 mg dose group, resulting in an objective response rate of 10%. Twenty-eight patients (45%) achieved stable disease (three in the brivanib alaninate 320 mg dose group, three in the brivanib alaninate 600 mg dose group and 22 in the brivanib alaninate 800 mg dose group), 15 patients (24.2%) had disease progression (two in the brivanib alaninate 320 mg dose group, two in the brivanib alaninate 600 mg dose group and 11 in the brivanib alaninate 800 mg dose group). Radiographic response was not evaluable in 13 patients (21%). The median duration of response was 9.2 months (95% CI: 7.20, 16.4 months; *n*=6), the median duration of disease control was 5.5 months (95% CI: 3.84, 7.32 months; *n*=28) and the median duration of stable disease was 5.3 months (95% CI: 3.68, 5.55; *n*=21). The median PFS was 3.9 months (95% CI: 3.38, 5.42 months; *n*=51).

### FDG-PET and *K-Ras* analyses

Results of FGD-PET and *K-Ras* analyses are reported in Appendix F.

## Discussion

This phase I study evaluated the safety, PK, pharmacodynamics and efficacy of brivanib combined with full-dose cetuximab in patients with advanced gastrointestinal malignancies. The MTD of the combination of cetuximab and brivanib was not established at the prespecified MDL of 800 mg, although this dose was determined to be the MTD for monotherapy in a previous phase I study (Jonker *et al*, 2010). One DLT occurred at the 320-mg dose level in one patient who experienced bilateral pulmonary artery emboli. The toxicity profile of brivanib alaninate in combination with cetuximab observed in this study was manageable.

The population in the current study was heavily pretreated, having advanced-stage disease; the majority of patients had received three or more previous lines of treatment and ∼40% of patients had prior EGFR inhibitor therapy. Despite this, brivanib alaninate in combination with cetuximab demonstrated promising antitumour activity, with an objective response rate of 10% at the MDL and a PFS of 3.9 months. Objective responses were not noted in the 11 patients treated with low-dose brivanib group, despite concomitant cetuximab therapy, possibly because of the low number of patients in this subgroup and the inclusion of patients with gastrointestinal malignancies other than colorectal cancer (where cetuximab does not have a proven efficacy as monotherapy), patients with unknown *K-Ras* status and patients who had received prior anti-EGFR therapies. Consistent with our findings, results from a phase III study of cetuximab monotherapy *vs* cetuximab plus irinotecan combination therapy in irinotecan-refractory patients showed a median PFS of 1.5 months with cetuximab monotherapy (Cunningham *et al*, 2004), and a separate study of cetuximab monotherapy in patients who were refractory or intolerant to both irinotecan and oxaliplatin by Jonker *et al* (2007) reported a median PFS of 1.9 months, with an overall survival of 6.1 months. Notably, the *K-Ras* wild-type subgroup in the study by Jonker's group had a response rate of 12.8% and overall survival of 9.5 months (Karapetis *et al*, 2008). In contrast, in the small subgroup of patients in the current study who had *K-Ras* wild-type status and did not receive prior anti-EGFR-targeted therapy, 6 of 11 patients had a partial response. However, it should be noted that these *K-Ras* analyses were unplanned and, therefore, limited. In a first-line setting, the combination of cetuximab with the VEGF-A-binding monoclonal antibody bevacizumab and oxaliplatin-based chemotherapy did not show a benefit for adding cetuximab in patients with *K-Ras* wild-type tumours. In patients with *K-Ras* mutant tumours, there was a detrimental effect (median PFS decreased from 12.5 to 8.1 months and overall survival decreased from 24.9 to 17.2 months) (Tol *et al*, 2009).

2[18F]Fluoro-2-deoxyglucose positron-emitting tomography is increasingly becoming an integral part of multicentre trials for tumour detection, staging and follow-up studies. In particular, changes in SUV parameters over the course of treatment can serve as an early surrogate marker for clinical benefit (Kelloff *et al*, 2005). The FDG-PET repeatability coefficients reported here indicate that 95% of patients had an SUV_max_ per cent difference in the two repeat baseline scans within −34% and 51%. Notably, the lower repeatability coefficient value of −34% rather than the −25%, as suggested by European Organisation for Research and Treatment of Cancer (EORTC) guidance, indicates a metabolic response by SUV_max_, while individual changes within −34% and 51% may not represent a metabolic response to treatment. The metabolic response rate, as determined by FDG-PET, was similar across all SUV parameters when assessed by the EORTC and lower repeatability coefficient threshold. At the 800-mg dose, metabolic response rate on day 15 (after 2 weekly doses of cetuximab and 1 week of continuous dosing of brivanib alaninate) was 53% by EORTC criteria and 43% by repeatability threshold. In addition, the metabolic response rate on day 56 was 39% by EORTC and 26% by repeatability threshold. Association of metabolic response and clinical outcome was assessed by SUV_max_, with results suggesting that metabolic response at days 15 and 56, based on the SUV_max_ parameter, may be associated with longer PFS in this population. In addition, greater separation of the Kaplan–Meier curves at day 56 than at day 15 may suggest that metabolic response at day 56 is a better predictor of PFS, a hypothesis that could be tested in future trials. Overall, these data support the concept that metabolic response may represent a predictive marker of clinical outcomes.

The promising efficacy observed in this study in patients with advanced gastrointestinal cancer and the manageable toxicity profile of brivanib justifies further study of brivanib in combination with cetuximab. A prospective, randomised, phase III study of brivanib alaninate plus cetuximab compared with placebo plus cetuximab in patients with advanced colorectal cancer whose tumours are *K-Ras* wild type and who have been previously treated with combination chemotherapy but are treatment naive for EGFR-targeted therapy has completed accrual February 2011 (the National Cancer Institute of Canada trial, clinicaltrials.gov identifier: NCT00640471); this study should help us better understand what additional benefit (if any) brivanib adds in combination with cetuximab in patients with advanced colorectal cancer.

## Figures and Tables

**Figure F1:**
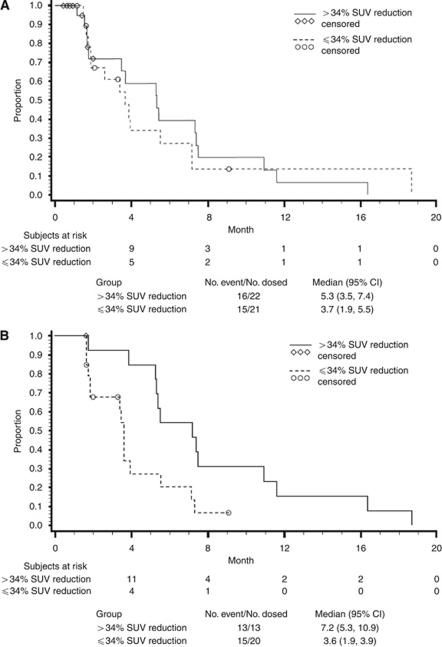
Kaplan–Meier plot of PFS by metabolic responder group FDG-PET repeatability threshold for metabolic changes at brivanib alaninate MTD (800 mg). (**A**) Day 15 and (**B**) day 56.

**Table 1:** (a) Baseline demographics and characteristics and (b) prior therapy

	**Brivanib alaninate dose, mg**			**Total**
	**320 (*n*=6)**	**600 (*n*=5)**	**800 (*n*=51)**	**(*N*=62)**
**(a)**				
Median age (min, max)	61 (50, 65)	59 (28, 74)	60 (31, 78)	60 (28, 78)
				
*Age,* n *(%)*				
≤65 years	5 (83)	4 (80)	36 (71)	45 (73)
⩾65 years	1 (17)	1 (20)	15 (29)	17 (27)
				
*Gender,* n *(%)*				
Male	3 (50)	2 (40)	33 (65)	38 (61)
Female	3 (50)	3 (60)	18 (35)	24 (39)
				
*Tumour type*				
Oesophagus	1 (17)		1 (2)	2 (3)
Colorectal	5 (83)	4 (80)	50 (98)	59 (65)
Other^a^	0 (0)	1 (20)	0 (0)	1 (2)

**(b)**	
**Prior therapy, *n* (%)**	**Total (*N*=62)**
Prior surgery	59 (95)
Prior radiotherapy	20 (32)
Prior hormonal, immunologic or biologic therapy	41 (66)
Prior VEGF-targeted therapy	36 (58)
Bevacizumab	34 (55)
VEGF trap	2 (3)
Prior EGFR-targeted therapy	27 (44)
Cetuximab	21 (34)
Panitumumab	6 (10)
Prior chemotherapy	59 (95)
	
*No. of regimens*	
1	10 (16)
2	15 (24)
⩾3	34 (55)

Abbreviations: EGFR=epidermal growth factor receptor; VEGF=vascular endothelial growth factor.

a Fibrolamellar liver tumour.

**Table 2:** Most common treatment-related (investigator determined) AEs and serious AEs

	**Brivanib alaninate dose, mg**			**Total (*N*=62)**	**Total (*N*=62)**
**AE**	**320 mg (*n*=6), *n* (%)**	**600 mg (*n*=5), *n* (%)**	**800 mg (*n*=51), *n* (%)**	**>15% incidence, any grade, *n* (%)**	**worst CTC grade** **, grade 3/4, *n* (%)**
Any AE	6 (100.0)	5 (100.0)	43 (84.3)	54 (87.1)	27 (43.5)
Dermatitis	1 (16.7)	1 (20.0)	14 (27.5)	16 (25.8)	2 (3.2)
Fatigue	3 (50.0)	2 (40.0)	30 (58.8)	25 (56.5)	8 (12.9)
Diarrhoea	1 (16.7)	2 (40.0)	18 (35.3)	21 (33.9)	1 (1.6)
Anorexia	1 (16.7)	1 (20.0)	19 (37.3)	21 (33.9)	2 (3.2)
AST increased	0	1 (20.0)	19 (37.3)	20 (32.3)	5 (8.1)
ALT increased	1 (16.7)	1 (20.0)	16 (31.4)	18 (29.0)	6 (9.7)
Vomiting	1 (16.7)	1 (20.0)	8 (15.7)	10 (16.1)	2 (3.2)
Headache	0	1 (20.0)	13 (25.5)	14 (22.6)	0 (0)
Mucositis	0	0	12 (23.5)	12 (19.4)	0 (0)
Nausea	1 (16.7)	1 (20.0)	10 (19.6)	12 (19.4)	1 (1.6)
Xerodermatitis	2 (33.3)	0	9 (17.6)	11 (17.7)	0 (0)
Hypertension	0	1 (20.0)	9 (17.6)	10 (16.1)	1 (1.6)
Pruritus	1 (16.7)	0	7 (13.7)	8 (12.9)	0 (0)
Proteinuria	0	1 (20.0)	7 (13.7)	8 (12.9)	0 (0)
					
**Serious AEs**	**320 mg** **(*n*=6), *n* (%)**	**600 mg** **(*n*=5), *n* (%)**	**800 mg** **(*n*=51), *n* (%)**	**Total** **(*N*=62) >4% incidence, any grade, *n* (%)**	
Disease progression	1 (16.7)	1 (20.0)	7 (13.7)	9 (14.5)	—
Vomiting	0	1 (20.0)	3 (5.9)	4 (6.5)	—
Dehydration	0	0	4 (7.8)	4 (6.5)	—
Pyrexia	0	0	3 (5.9)	3 (4.8)	—

Abbreviations: AE=adverse events; ALT=alanine transferase; AST=aspartate aminotransferase; CTC=common toxicity criteria.

**Table 3:** Effect of cetuximab on brivanib pharmacokinetics following single 800 mg doses of brivanib alaninate with cetuximab 400 mg m^–2^ on day 8 and without cetuximab 400 mg m^–2^ on day 1

**Treatment and comparison**	***C*_max_ (ng ml^–1^) geometric mean (CV)**	**AUC(INF) (ng h ml** ^–1^ **) geometric mean (CV)**
Day 1: brivanib alaninate	3580.1 (42)^a^	45620.2 (44)^b^
Day 8: brivanib alaninate plus cetuximab	3233.3 (50)^c^	42913.6 (58)^d^
Day 8: day 1 geometric mean ratio point estimate (CI)	0.903 (0.803–1.016)	0.933 (0.838–1.039)

Abbreviations: AUC(INF)=area under the plasma concentration–time curve extrapolated to infinity; *C*_max_=maximum plasma concentration; CI=confidence interval; CV=coefficient of variation.

a *n*=50.

b *n*=48.

c *n*=49.

d *n*=4.

**Table F1:** (a) FDG-PET reproducibility and other summary statistics for baseline SUV parameters (*N*=51). (b) Frequency of FDG-PET metabolic responders in patients receiving the MDL (800 mg) in combination with cetuximab (*N*=53)

**(a)**					
	***N*=53 evaluable**				
**SUV parameter**	**Summary across lesions**	**Intrapatient CV (%)**	**Mean per cent difference point estimate (95% CI; %)** a	**95% Repeatability coefficients (%)**	
SUV_max_	Average	15.9	1.9 (–3.3, 7.3)	–33.6	50.1
	Max	16.4	1.4 (–3.9, 7.0)	–34.3	52.3
SUV_mean_	Average	14.9	0.2 (–4.6, 5.3)	–31.9	46.8
	Max	15.4	1.2 (–3.9, 6.4)	–32.8	48.9
SUV_peak_	Average	17.0	2.2 (–3.3, 8.1)	–35.2	54.3
	Max	17.3	2.0 (–3.6, 8.0)	–35.7	55.6

**(b)**				
	**Metabolic response criteria (*N*=53 evaluable)**			
**SUV**	**EORTC criteria (>25% reduction *n*, %)**		**RC** **criteria (SUV reduction threshold**^b^ ***n*, %)**	
**parameter**	**Day 15**	**Day 56**	**Day 15**	**Day 56**
SUV_max_	27 (53)	20 (39)	22 (43)	13 (26)
SUV_mean_	27 (53)	19 (37)	21 (41)	17 (33)
SUV_peak_	28 (55)	20 (39)	20 (39)	14 (27)

Abbreviations: CI=confidence interval; CV=coefficient of variation; EORTC=European Organisation for Research and Treatment of Cancer; FDG-PET=2[18F]fluoro-2-deoxyglucose positron-emitting tomography; MDL=maximum dose level; RC=repeatability coefficients; SUV=standardised uptake value.

a Percent difference of second from first scan based on geometric means from log-transformed data.

b SUV_max_ >34% reduction, SUV_mean_ >32% reduction, SUV_peak_ >35% reduction.Percentages based on all patients with baseline SUV measurements.

**Table F2:** Frequency of tumour *K-Ras* mutation status by (a) best ORR and (b) best ORR and prior anti-EGFR therapy (brivanib alaninate 800 mg dose group)

**(a)**				
	**Partial response *n* (%)**	**Stable disease**^a^ ***n* (%)**	**Progressive disease *n* (%)**	**Total, *n***
*K-Ras* mutant	0 (0)	11 (73.3)	4 (26.7)	15
*K-Ras* wild type	6 (31.6)	8 (42.1)	5 (26.3)	19
				
**(b)**				

*K-Ras* mutant	0 (0)	7 (63.6)	4 (36.4)	11
*K-Ras* wild type	6 (54.55)	3 (27.3)	2 (18.2)	11
				
*Prior anti-EGFR therapy*				
*K-Ras* mutant	0 (0)	4 (100)	0 (0)	4
*K-Ras* wild type	0 (0)	5 (62.5)	3 (37.5)	5

Abbreviations: EGFR=epidermal growth factor receptor; ORR=overall response rate.

a ⩾6 weeks.

## APPENDICES

### Appendix A – Treatment

Dose modifications of brivanib alaninate and/or cetuximab were made in response to AEs in individual patients. A minimum of three patients were treated at each dose level. If a DLT was observed in one of three patients at a given dose level, an additional three patients were enrolled at that dose level before further escalation was considered. At any dose level, up to four additional patients could be enrolled (to a maximum of 10 patients per cohort) to obtain additional safety or efficacy data. The MTD of the continuous daily dose schedule of brivanib alaninate in combination with cetuximab was defined as the highest dose of brivanib alaninate at which one-third or fewer of the patients experienced a DLT. Once the MTD was determined, an expansion cohort was to be evaluated at that dose; however, the MTD was not formally reached and the expansion cohort was opened at the predefined MDL based on the MTD (800 mg) reached in the phase I brivanib monotherapy study (CA182002) (Jonker *et al*, 2010).

In the dose-expansion phase, only patients with histologic verification of colorectal cancer were enrolled. In order to have 40 response-evaluable patients at the MDL, at least 50 patients with advanced colorectal cancer were to be enrolled.

### Appendix B – Analyses of cetuximab and brivanib concentrations in serum and plasma

Serum cetuximab concentrations were measured by enzyme-linked immunosorbent assay with colorimetric detection employing a recombinant human EGFR full-length receptor adsorbed onto a microtitre plate to capture cetuximab in 0.1% human serum. The captured cetuximab was detected using a commercial rabbit-antihuman IgGFC-HRP conjugate. The limits of quantitation ranged from 1 to 32.0 μg ml^–1^.

Brivanib concentrations were measured in glycinated plasma, after solid-phase extraction by a liquid chromatography/tandem mass spectrometry method using a stable-labelled internal standard of brivanib ([13C3,15N2]BMS-540215). The internal standard (50 μl at 100 ng ml^–1^ for plasma) was added to 0.1 ml of plasma sample and the samples buffered with ammonium formate before loading onto solid-phase extraction plates (Waters Corporation, Milford, MA, USA) conditioned with methanol and an ammonium formate solution. After addition of the plasma sample, the plates were washed with an ammonium formate solution and acetonitrile/water. Samples were eluted from the plates with methanol containing formic acid and evaporated to dryness under nitrogen, and then reconstituted with an acetonitrile/ammonium formate solution. The high-performance liquid chromatography (HPLC) system consisted of a Shimadzu HPLC pump (Shimadzu Corporation, Columbia, MD, USA), an LEAP PAL Autosampler (LEAP Technologies, Carrboro, NC, USA) and a Phenomenex column (Torrance, CA, USA) Luna Phenyl-Hexyl, 5 μm 2.0 × 50 mm^2^. The mobile phase flow rate was 0.3 ml min^–1^, and a gradient of two solvent systems, A and B, was used for HPLC profiling. Solvent A consisted of 0.01 M ammonium formate solution in water. Solvent B consisted of 0.01 M ammonium formate in acetonitrile/water. The HPLC system was interfaced to a Sciex API 3000 mass spectrometer (Applied Biosystems, Foster City, CA, USA) operated in the positive ion electrospray ionisation mode. The total run time was ∼6.0 min and the retention time for brivanib and the internal standard were ∼2.5 min, with limits of quantitation ranging from 2 to 2000 ng m^–1^.

### Appendix C – Efficacy analyses

Tumour measurements were obtained at screening (within 28 days prior to the start of treatment), within 7 days of the start of every second cycle of treatment (i.e. every 8 weeks), and at the end of treatment. Efficacy end points included best overall clinical response, PFS, ORR, disease control rate and prolonged disease control (PDC, defined as a best overall response of complete response, partial response or stable disease of at least 120 days) rate, duration of response and duration of disease control. Patients who never received treatment were not evaluable for response and patients meeting the criteria for a complete or partial response had confirmatory tumour measurements obtained between ∼4 and 6 weeks of initially demonstrating a response.

### Appendix D – FDG-PET and *K-Ras* analyses

#### FDG-PET analysis

2[18F]fluoro-2-deoxyglucose positron-emitting tomography analyses were performed as described previously (Velasquez *et al*, 2009). In brief, FDG-PET scans were obtained on two separate days within 14 days before day 1 (double baseline scans) and then once on or around days 15 and 56 (prior to cycle 3). Metabolic changes in tumour by FDG-PET were measured by the SUV parameters SUV_max_, SUV_mean_ and SUV_peak_, each calculated as the sum of the parameter's value across lesions.

#### Predictive biomarkers

*K-Ras* mutation status was assessed by sequencing of codons 12 and 13 from DNA isolated from paraffin-embedded tumour tissue from colorectal cancer patients, as reported previously (Karapetis *et al*, 2008).

### Appendix E – Statistical analyses

Based on these two-stage approach, to determine whether a 15% ORR was likely, 19 patients were accrued in the first stage. If no responses were observed, it was concluded that the true response rate was unlikely to be ⩾15% and no more patients were enrolled at this dose. If there was at least one response observed in the first stage, 21 additional patients were enrolled at the MDL. With this sample size, there was no >5% chance of declaring that there was no therapeutic effect when actually there was an effect. With a total of 40 response-evaluable patients, the maximum width of the two-sided 95% CI for ORR would be 25% when the ORR was in the expected 15±12.5% range. To assess the effect of the combination of brivanib alaninate and cetuximab at the MDL on the PK of brivanib, point estimates and 90% CIs for the ratios of the geometric means of brivanib *C*_max_ AUC(INF) following administration of brivanib alaninate with and without cetuximab were constructed. The estimates were generated using a general linear model on log(*C*_max_) and log(AUC(INF)) for patients treated at the MDL, with treatment as a fixed effect and measurements within each patient as repeated measurements. Point estimates and 90% CIs for means and treatment differences on the log scale were exponentiated to obtain estimates for *C*_max_ and AUC(INF) geometric means and ratios of geometric means on the original scale. Summary statistics were tabulated for the brivanib PK parameters *C*_max_ and AUC(INF) by brivanib alaninate dose and study day. For FDG-PET imaging biomarker analysis, repeatability of SUV parameters was assessed by repeatability coefficients for SUV parameters summarised by mean and maximum across lesions. The mean (or mean per cent) baseline difference in each SUV parameter was estimated by 95% CI for each parameter, and the intrapatient coefficient of variation was calculated. Summary statistics for SUV_mean_, SUV_peak_ and SUV_max_ (summed across lesions) and changes from baseline were tabulated by brivanib alaninate dose and FDG-PET scan visit (days 15 and 56). The frequency of metabolic responders on days 15 and 56, based on the EORTC criteria and on thresholds identified by the repeatability analysis, was tabulated by brivanib alaninate dose for each parameter. To explore associations between FDG-PET metabolic changes by SUV_max_ and clinical outcome (PFS), point estimates, and 95% CIs were calculated for median PFS for metabolic responders and nonresponders using Kaplan–Meier methodology. For *K-Ras* mutation analysis, Fisher's exact test was used to assess association between the mutation status and PDC status. In addition, a logistic regression model was fit to model the probability of disease control as a function of *K-Ras* mutation status. A Cox proportional hazards model was used to assess the relationship between PFS and *K-Ras* mutation status. Associations were summarised in terms of point and interval estimates of odds ratio (OR) and hazard ratios (HRs). The Kaplan–Meier product-limit method was used to plot PFS by mutation status. *K-Ras* mutation analyses were performed only in the 800-mg (MDL) cohort, as 800 mg was the dose selected for further clinical studies.

### Appendix F – Results of FDG-PET and K-*Ras* analyses

#### FDG-PET

Repeatability coefficients indicated that 95% of the patients had an SUV_max_ per cent difference in the two repeat baseline scans within –34% and 52% (Table 1a). Current EORTC guidelines state that a metabolic response is defined as a reduction of at least 25% in SUV parameters from baseline (Young *et al*, 1999). Based on the repeatability evaluation in this study, metabolic response was determined using both the EORTC threshold and the repeatability coefficient threshold of at least a 34% reduction in SUV_max_. Metabolic responses at the MDL were similar across all SUV parameters on each study day (Table 1b). The metabolic response rate based on the EORTC guidelines was 53% on day 15 and 39% on day 56. However, the metabolic response rate was somewhat lower when using the repeatability thresholds, that is for SUV_max_ it was 43% on day 15 and 26% on day 56. By either criterion (EORTC guidelines or repeatability thresholds), fewer patients showed a metabolic response on day 56 compared with day 15, with 8 patients nonevaluable on day 15 and 18 patients nonevaluable on day 56. The frequency of metabolic responses at the lower doses was similar, although this was based on much smaller sample sizes. In the 800-mg cohort, the median PFS among the metabolic responders using the repeatability coefficient threshold of at least a 34% reduction in SUV_max_ was longer than median PFS among metabolic nonresponders on both days 15 and 56, although the difference was less pronounced on day 15 (Figure F1).

#### Predictive biomarkers

Tumour *K-Ras* mutation analysis was assessed in 45 patients in the brivanib alaninate 800 mg cohort, 34 of whom had data on best overall response rate, and 30 of whom had data on PDC. No patients with a *K-Ras* mutant tumour achieved a radiographic partial or complete response. On the other hand, 31.6% (6 out of 19) patients with a *K-Ras* wild-type tumour had a partial response (Table F1a). In all, 11 of 15 patients with a *K-Ras* mutant tumour (73.3%) had stable disease (⩾6 weeks), while 8 of 19 (42.1%) patients with a *K-Ras* wild-type tumour had stable disease (Table F1a); and 6 of the 11 (55%) patients who had not received prior anti-EGFR therapy had a partial response (Table F2b) and 9 (82%) had PDC (>120 days).

Prolonged disease control was observed in 25% (3 out of 12) of patients with a *K-Ras* mutant tumour and in 66.7% (12 out of 18) of patients with a *K-Ras* wild-type tumour (uncorrected *P=*0.06 from Fisher's exact test; *P=*0.02 from logistic regression model, OR [95% CI]=0.17 [0.03, 0.79]). Median PFS was longer in patients with *K-Ras* wild-type tumours than those with *K-Ras* mutant tumours (218 *vs* 112 days, respectively; from the Cox proportional hazards model, *P=*0.01, HR [95% CI]=2.82 [1.25, 6.36]).
